# P-1464. Clinical Outcomes of patients with Infection Secondary to VancomycinResistant Enterococci (VRE)

**DOI:** 10.1093/ofid/ofae631.1636

**Published:** 2025-01-29

**Authors:** Anood Alqura’an, Zaid Al khouri, Mollie VanNatta, Ruhul Munshi, Alexandre Malek

**Affiliations:** LSU HEALTH SHREVEPORT, shreveport, Louisiana; Louisiana State University Health Sciences Center Shreveport, Shreveport, Louisiana; Ochsner LSU Health Shreveport, Shreveport, Louisiana; LSU Health Shreveport, Shreveport, Louisiana; LSU Health shreveport, Shreveport, Louisiana

## Abstract

**Background:**

Daptomycin dose dependent VRE infection is a growing clinical challenge to patients and healthcare providers. In this study, we sought to describe patients with infections secondary to Daptomycin-susceptible and dose-dependent VRE.

**Figure:**
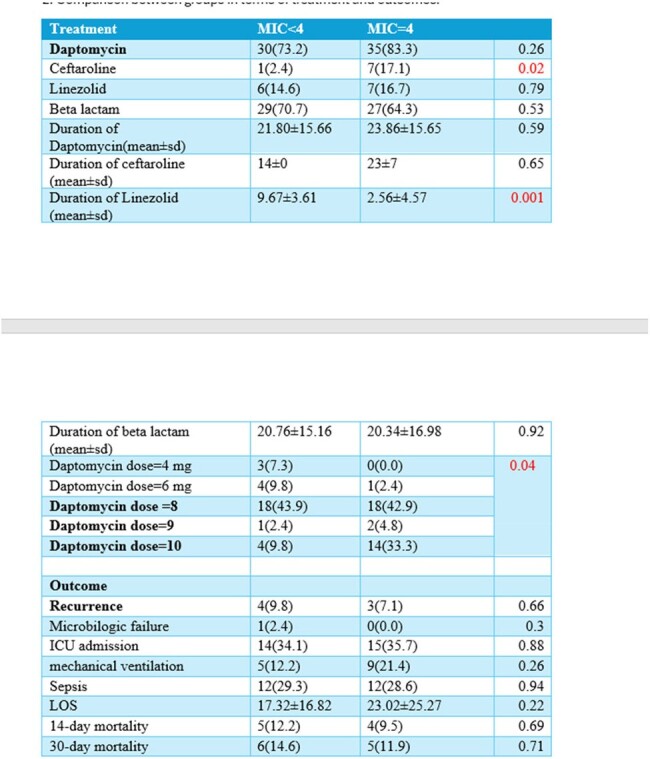

**Methods:**

We conducted a retrospective cohort study of adult patients admitted at Ochsner LSU Health Shreveport- Academic Medical Center who were diagnosed with VRE infections between 2019 and 2024. We evaluated risk factors, comorbidities, treatment regimens and clinical outcomes of VRE infections with daptomycin MIC 4 mcg/ml (group 1) vs those with VRE daptomycin MIC < 4 mcg/ml (group 2).

**Figure:**
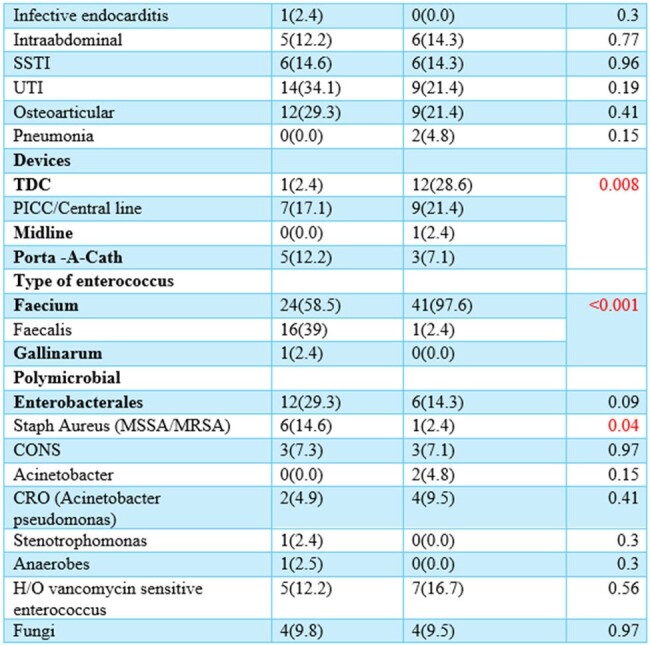

**Results:**

Out of 85 patients with different VRE infections, 42 (49.41%) had a daptomycin MIC = 4, while 41 (48.23%) had a daptomycin MIC < 4. Different sources of VRE infections were identified among the two groups. Polymicrobial infection rates were 54.76% in group 1 and 60.97% in group 2. History of previous infection with vancomycin-sensitive Enterococcus was observed in 12% of group 1 and 17% of group 2. The majority of Enterococcus isolates were E. faecium: 97.6% in group 1 and 58.5% in group 2. Comparable factors between the two groups included the presence of external lines, coexisting chronic kidney disease (CKD), and previous beta-lactam use. The targeted antibiotics included daptomycin only, linezolid only, or a combination of either one with ceftaroline or a beta-lactam. The duration of daptomycin treatment was 23.86 days in group 1 and 21.8 days in group 2. In group 1, 80.95% received a daptomycin dose higher than the standard dose of 6 mg/kg, compared to 46.34% in group 2. A dose of 10 mg/kg was significantly observed in 33.3% of group 1 and 9.8% of group 2. The 30-day mortality rate in group 1 (MIC = 4) was 11.90%, whereas in group 2 (MIC < 4), it was 14.63%.

**Figure:**
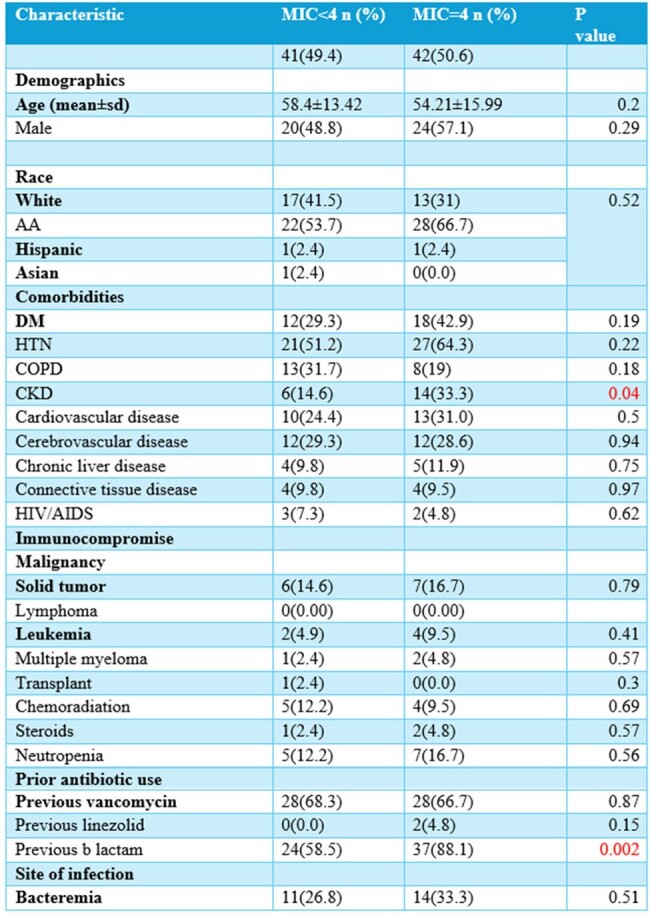

**Conclusion:**

Our data revealed a higher incidence of VRE infections with a MIC = 4 mcg/ml among Enterococcus faecium species. Additionally, VRE infections with a higher MIC = 4 were associated with comorbidities such as chronic kidney disease and the presence of external devices and central venous catheters. Understanding these risk factors can aid in identifying high-risk patient populations and implementing targeted preventive measures.

**Disclosures:**

**All Authors**: No reported disclosures

